# Public Health Nursing in the 21st Century: Challenges and Opportunities for Women and Children's Health

**DOI:** 10.1155/2013/939230

**Published:** 2013-11-03

**Authors:** Sue Peckover, Sophie Mogotlane, Kari Glavin, Megan Aston

**Affiliations:** ^1^Centre for Applied Childhood Studies, University of Huddersfield, Queensgate, Huddersfield HD1 3DH, UK; ^2^University of South Africa, Pretoria, South Africa; ^3^Department of Nursing, Diakonova University College, Fredensborgveien 24 Q, 0177 Oslo, Norway; ^4^School of Nursing, Dalhousie University, Halifax, Nova Scotia, Canada B3H 4R2

This special issue focuses upon public health nursing with women and children in the 21st century. There are 6 papers which address a range of topics illustrating some of the opportunities and challenges arising in this area of nursing work from Ireland, Norway, Finland, UK, Canada, and Brazil.

Two papers (A. Clancy et al. and R. Bryars et al.) remind us of how public health nursing varies according to context and population. This is illustrated in a comparative sense in the paper by A. Clancy and colleagues which compares public health nursing models in Ireland and Norway. These two countries have many similarities from a geographic and demographic perspective. Both have similar sized populations, but economically there are vast differences between Ireland and Norway in relation to poverty; most notably, life expectancy is lower and inequalities are higher in Ireland. Nevertheless, both countries have a strong commitment to WHO reforms towards primary care, and public health nurses have been identified as key players in the delivery of primary care services, particularly primary prevention. Families with children living in areas of high deprivation face multiple health and social challenges and this high level of need impacts on the work of health practitioners working in such areas. The paper by R. Bryar and colleagues reports the use of a Delphi approach to identify priority areas for health visiting practice in an area of deprivation in the UK. This process identified a wide spectrum of health and social needs indicative of the level of deprivation in the area and pressures on families with children and is the first stage of a six-stage project which aims to develop a toolkit of resources for evidence-based practice in health visiting.

Public health nursing is often focused on addressing the health needs of “at risk” or marginalized groups, and this is illustrated in different ways in 3 papers which address postnatal depression (K. Glavin and P. Leahy-Warren), child maltreatment (J. Inkilä and colleagues), and maternal substance abuse (N. Letourneau and colleagues). K. Glavin, and P. Leahy-Warren discuss the global prevalence of postnatal depression and how this serious health issue is specifically addressed in Ireland and Norway. They cite international research studies that clearly indicate the positive difference public health nurses make when screening and referring for postnatal depression. Although the health care systems are dramatically different between Ireland and Norway, both countries strive to offer universal services to all new mothers by public health nurses.

Child maltreatment is a global problem and a multi-dimensional phenomenon occurring in all social classes. The paper by J. Inkilä and colleagues depicts interprofessional collaboration associated with the detection of and early intervention in child maltreatment taking place in the family in Finland. The results provide basic knowledge of interprofessional collaboration associated with child maltreatment between the agencies involved in the study. The research evidence can be utilized in an international context when developing collaboration between different fields. Protecting children from exposure to maternal substance abuse is a public health priority and is addressed in the paper by N. Letourneau and colleagues. Their study examines mothers' experiences and engagement with a community based methadone treatment programme in Canada. It highlights practical barriers to engagement such as transport and childcare as well as the importance of including child and family-focused interventions in order to strengthen parenting capacity and promote the wellbeing of both mothers and their children.

The paper by C. A. Bonow and colleagues shifts attention towards another aspect of Public Health Nursing. It focuses on occupational health in Brazil and comprehensively addresses issues of occupational health risks in the welding industry generally and specifically as these apply to women. These risks are numerous and include noise, accidents such as burns and explosions, respiratory problems linked to the gases, welding fumes and dust, and back pain and physical exhaustion due to long standing sometimes in abnormal postures. The paper also begins to create an awareness of these health risks and discusses preventive measures such as hand washing and limited exposure in terms of breaks in the day and leave. 

The papers in this special issue provide an overview of the range and scope of public health nursing work with women and children. Although similar health issues exist around the world for women and children, public health nurses continue to create and offer programmes and services that are responsive to the unique needs of “at risk” populations in their own countries. The debate and challenge about universal and targeted programs is a common topic in some of the papers and is addressed through different forms of assessment and programming for mothers and families. Accessibility to programmes is another common global theme found throughout the papers. For example, Norway and Ireland have different programs to ensure accessibility for “at risk” mothers, and the methadone clinic in Canada clearly details the challenges of accessibility for clients due to their social circumstances. The importance of well-organized institutional practice models to support the work of public health nurses is another global issue that is shared by our authors. The Delphi model is offered as one way to assist public health nurses to effectively support families experiencing high deprivation, and other authors talk about how an interprofessional collaboration model can be used to address the maltreatment of children. Across all of the papers, it becomes clear that there are multiple roles for public health nurses when working with women and children. The strength of public health nurses is their ability to work with “at risk” populations at personal, programme, and institutional levels within public health units or private businesses. However, ongoing challenges include debates about best practices. The papers presented in this special issue provide up-to-date evidence with regard to how public health nurses are effectively addressing the diverse health needs of women and children around the world. 

*Megan Aston* *Megan Aston* *Kari Glavin* *Kari Glavin* *Sophie Mogotlane* *Sophie Mogotlane* *Sue Peckover* *Sue Peckover*

